# Mechanisms and applications of radiation-induced oxidative stress in regulating cancer immunotherapy

**DOI:** 10.3389/fimmu.2023.1247268

**Published:** 2023-08-04

**Authors:** Zhuangzhuang Zheng, Jing Su, Xueying Bao, Huanhuan Wang, Chenbin Bian, Qin Zhao, Xin Jiang

**Affiliations:** ^1^ Jilin Provincial Key Laboratory of Radiation Oncology & Therapy, The First Hospital of Jilin University, Changchun, China; ^2^ Department of Radiation Oncology, The First Hospital of Jilin University, Changchun, China; ^3^ National Health Commission (NHC) Key Laboratory of Radiobiology, School of Public Health of Jilin University, Changchun, China

**Keywords:** radiotherapy, reactive oxygen species, oxidative stress, tumor immune microenvironment, immunotherapy

## Abstract

Radiotherapy (RT) is an effective treatment option for cancer patients, which induces the production of reactive oxygen species (ROS) and causes oxidative stress (OS), leading to the death of tumor cells. OS not only causes apoptosis, autophagy and ferroptosis, but also affects tumor immune response. The combination of RT and immunotherapy has revolutionized the management of various cancers. In this process, OS caused by ROS plays a critical role. Specifically, RT-induced ROS can promote the release of tumor-associated antigens (TAAs), regulate the infiltration and differentiation of immune cells, manipulate the expression of immune checkpoints, and change the tumor immune microenvironment (TME). In this review, we briefly summarize several ways in which IR induces tumor cell death and discuss the interrelationship between RT-induced OS and antitumor immunity, with a focus on the interaction of ferroptosis with immunogenic death. We also summarize the potential mechanisms by which ROS regulates immune checkpoint expression, immune cells activity, and differentiation. In addition, we conclude the therapeutic opportunity improving radiotherapy in combination with immunotherapy by regulating OS, which may be beneficial for clinical treatment.

## 1 Introduction

Immunotherapy, a treatment that uses the immune system to eliminate cancer cells, has been touted as a potential cure for cancer in recent years (1). Immunotherapy eliminates cancer cells in the body by enhancing the recognition of tumor-associated antigens (TAAs) and specific killing effect of immune system (2). However, due to the heterogeneity of the tumor microenvironment (TME) and the immunosuppressive response of tumor cells, most patients do not benefit from the immunotherapy regimen.

Radiotherapy (RT) is a critical nonsurgical treatment for cancer. RT usually induces cell death by increasing the level of reactive oxygen species (ROS) in tumor cells (3). RT causes the production of a variety of ROS in tumor cells, which is one of the main ways of radiation-induced DNA damage and cell death (4). RT can directly induce DNA base damage. RT can also trigger ionization of water molecules, resulting in the production of large amounts of free radicals and ROS that damage DNA, lipids and proteins, leading to metabolic and functional changes and ultimately apoptosis (5). In addition, RT can favorably modulate immunological response, leading to increased tumor antigen presentation, priming of tumor-specific cytotoxic T cells, as well as enhanced T-cell homing, engraftment, and function in tumors (6).

**Figure 1:**
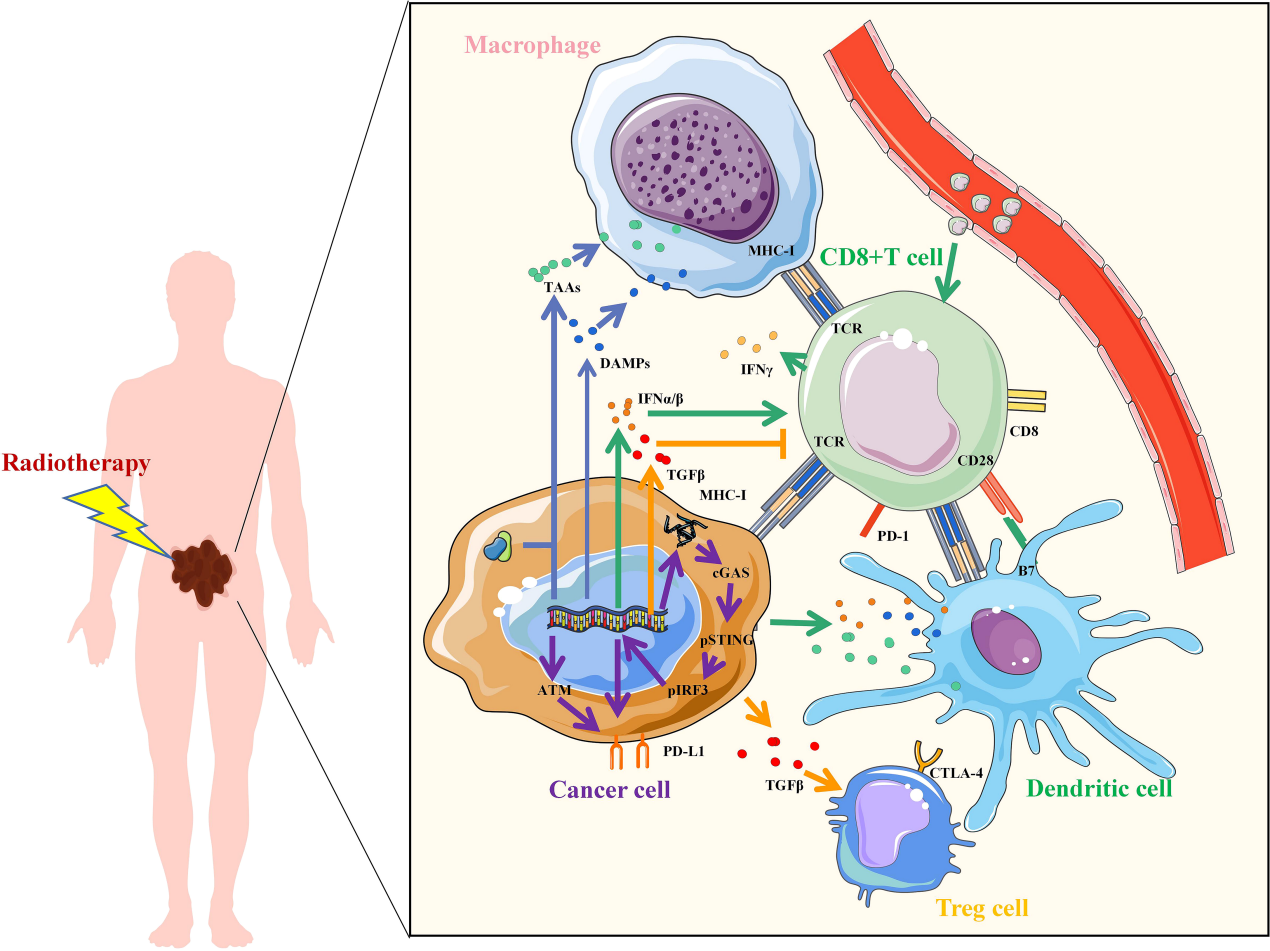
Mechanisms of radiotherapy to enhance immunotherapy. Irradiation increased the upregulation of tumor antigens, TAAs, tumor-associated antigens; DAMPs, damage-associated molecular patterns; IFN, interferon; TGF, transforming growth factor; MHC, major histocompatibility complex; TCR, T cell receptor; CD, Cluster of Differentiation; PD-L1, programmed cell death-ligand 1; PD-1, Programmed death 1; B7, CD80 and CD86; CTLA-4, cytotoxic T lymphocyte-associated antigen-4; cGAS, cyclic GMP-AMP synthase; STING, stimulator of interferon genes; IRF3, interferon regulatory factor 3; ATM, ataxia telangiectasia mutated protein.

Radiotherapy can enhance the effect of immunotherapy through a variety of mechanisms (**Figure 1**). Firstly, irradiation (IR) can increase the recognition of immune cells. IR damages DNA and proteins directly or indirectly through free radical production, which leads to an increase in neoantigens released by tumor cells for immune recognition. Tumor cells also release damage-associated molecular patterns (DAMPs) after IR, including high-mobility group box 1 (HMGB1), heat shock proteins (HSPs), and calreticulin (CRT), which mediate phagocytosis of antigen-presenting cells (APCs) and initiate tumor-specific T cell activation. In addition, T cell activation is mediated by the recognition and binding of major histocompatibility complex (MHC) molecules to which T cell receptor (TCR) bind peptides. IR can increase the expression of MHC-I in tumor cells, making it easier for cytotoxic T cells to recognize. Secondly, IR activates innate immune response and immune checkpoint upregulation. activation of the stimulator of interferon genes (STING) is an important part of innate immune response. The damaged DNA fragments after IR are released into the cytoplasm and recognized by cyclic GMP-AMP synthase (cGAS) to synthesize cyclic GMP-AMP (cGAMP), which induces the production of type I interferon (IFN) through the stimulation of STING-TBK1-IRF3 signal axis. Type I IFN regulates dendritic cells (DCs) function and helper T cell differentiation, mediating innate immune response. However, STING may also induce IR resistance and immunosuppression. On the one hand, STING can induce up-regulation of programmed cell death-ligand 1 (PD-L1) to promote immune escape. On the other hand, STING can promote IR and immune resistance of tumors through myeloid-derived suppressor cells (MDSCs) mobilization and indoleamine 2,3-dioxygenase (IDO) activation. IR-induced DNA damage can also directly up-regulate the expression of PD-L1 on tumor cells through ATM/ATR/Chk1 kinase, or indirectly regulate the expression of PD-L1 by increasing the secretion of IFN-γ. Thirdly, radiation-regulated immune microenvironments. IR can increase CD8^+^T cell infiltration and IFN-γ, promote the normalization of tumor vasculature and induce the polarization of M2-like macrophages towards M1 phenotype. However, IR can also promote the secretion of transforming growth factor β (TGF-β), inhibit CD8^+^T cells and increase the proportion of regulatory T cells (Tregs), leading to immunosuppression in tumors. To enhance the antitumor effect, the combination of RT and immunotherapy has become a new strategy for cancer care.

Recent evidence suggests that ROS-induced oxidative stress (OS) can regulate multiple tumor immune responses. ROS plays a mediator role of pivotal functions such as phagocytosis, antigen presentation and recognition, cytolysis and phenotypical differentiation in immune cells (7). Moderate level of ROS contribute to activation and differentiation of T cells, whereas high level of ROS impair T cell survival and function (8). Tumor-associated macrophage (TAM), MDSCs and Tregs are the main immunosuppressive cells in TME (9). ROS is an important regulator of their immunosuppressive function. Increased ROS level in TME may promote the differentiation of TAM into M2 subtypes and regulate PD-L1 expression (10). Tregs are less susceptible to cell death induced by OS compared to other CD4^+^T cells and ROS may promote the expression of forkhead box protein 3 (FOXP3) in Tregs and maintain the immunosuppressive function (11, 12). ROS is essential for MDSCs in their undifferentiated state. The release of ROS molecules mediates the suppression of T cells and the activation of Tregs (9, 13). Production of several immunosuppressive cytokines is also regulated by ROS, such as IL-6, IL-10 and TGF-β, which can block the function of immune cells (14, 15). In addition, increased ROS can mediate the expression of immune checkpoints such as PD-L1 to promote tumor immune escape (16). Given the regulatory role of ROS in antitumor immunity, it is necessary to summarize the role of IR-induced OS in cancer immunotherapy.

In this review, we summarize several mechanisms by which RT induces ROS production and OS in tumors. The molecular mechanism underlying the interaction between OS and anti-tumor immunity is further discussed, with emphasis on the regulatory role of ROS on TME. On this basis, we attempted to find a treatment method for regulating OS during RT to enhance the efficacy of radiotherapy combined with immunotherapy.

## 2 IR-induced OS promotes tumor cell death

**Figure 2:**
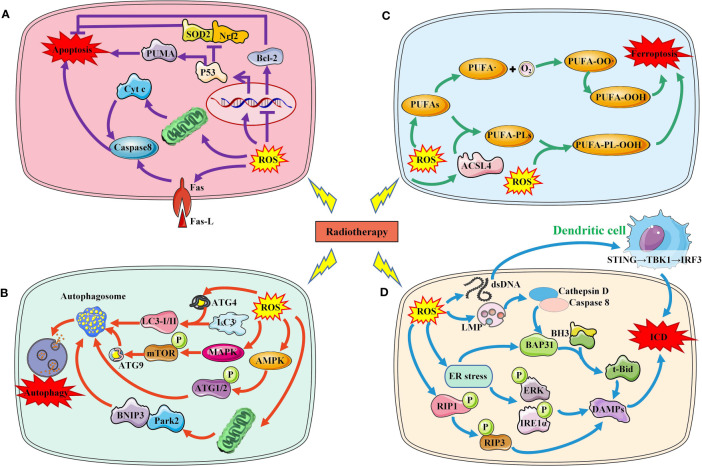
Several major ways in which RT-induced ROS induce tumor cell death. **(A)**. RT-induced ROS induces tumor cell apoptosis through P53 pathway, mitochondrial pathway and death receptor ligand pathway. **(B)** RT-induced ROS induces autophagy of tumor cells by activating AMPK, MAPK and LC3. **(C)** RT-induced ROS increased lipid peroxidation induced tumor cell ferroptosis. **(D)** RT-induced ROS increased ICD by increasing DAMPs production and immune cell activation.SOD2, superoxide dismutase 2; Nrf2, nuclear factor‑erythroid 2 related factor 2; PUMA, p53 upregulated modulator of apoptosis; Bcl-2, B-cell lymphoma-2; Cty c, Cytochrome C; ROS, reactive oxygen species; ACSL4, acyl-CoA synthetase long-chain family member 4; PUFAs, polyunsaturated fatty acids; LMP, Lysosome membrane permeability; BH3,; BAP31, BCR associated protein Bap 31; ER, endoplasmic reticulum; RIP, receptor interacting protein; ERK, extracellular regulated protein kinases; IRE1, Inositol-requiring enzyme 1; DAMPs, damage-associated molecular patterns; ICD, immunogenic cell death; ATG, autophagy protein; MAPK, mitogen-activated protein kinase; AMPK, adenosine 5`-monophosphate-activated protein kinase; mTOR, mechanistic target of rapamycin; BNIP3, BCL2/adenovirus E1B 19kDa interacting protein 3; Park2, Recombinant Parkinson Disease Protein 2.

Increased ROS is a critical way to kill tumor cells exposed to RT. After exposure, water molecules are broken down to produce large amounts of ROS, including the superoxide anion, Hydroxyl agent and hydrogen peroxide, respectively (17). RT-induced ROS promotes the death of tumor cells mainly by damaging macromolecular substances such as DNA, lipids, and proteins in cells. When RT-induced ROS level exceed their own antioxidant level, tumor cells death will occur in a variety of ways, including apoptosis, autophagy, and ferroptosis and others. In addition, injured and dead tumor cells activate the anti-tumor immune mechanism and induce immunogenic death of tumor cells (**Figure 2**).

### 2.1 Apoptosis and autophagy

DNA is one of the main targets of ROS, which can induce DNA damage and induce DNA damage response (18). When the level of ROS-induced DNA damage exceeds the cell’s ability to repair it, the cell initiates the apoptosis program. P53 is an important molecule that regulates apoptosis. Increased ROS can lead to p53 activation and its downstream upregulation of p53 upregulated modulator of apoptosis, the pro-oxidant genes (19). Furthermore, ROS-induced p53 has been demonstrated to downregulate antioxidant proteins and the anti-oxidant transcriptional factor, including superoxide dismutase 2 and nuclear factor−erythroid 2 related factor 2 (Nrf2) (20, 21). Caspases are a family of proteases in cells, activated by death receptor-dependent pathway and mitochondrial-dependent pathway. ROS can cause the activation of caspase 8 by regulating the expression of the death receptors and its ligand such as Fas and Fas L. Another way of apoptosis is mediated by mitochondria. ROS can not only damage mitochondrial DNA, but also damage mitochondrial electron transport chain, causing mitochondrial dysfunction. Mitochondrial dysfunction results in the release of proapoptotic protein cytochrome c and activates caspases to induce apoptosis. B-cell lymphoma-2 (Bcl-2) is a key anti-apoptotic protein molecule in apoptosis cells. ROS can down-regulate the expression of Bcl-2 and promote apoptosis through oxidative modification.

Autophagy is another important form of RT-induced tumor cell death. ROS accumulation induced by IR can regulate autophagy through a variety of pathways. Autophagy protein (ATG)4 is the core of autophagy regulation, and ROS can directly oxidize ATG4, leading to the accumulation of autophagosomes. ROS can also activate adenosine 5’-monophosphate-activated protein kinase signaling and induce autophagy initiation through ATG1 complex. Similarly, radiation-induced ROS can also activate mitogen-activated protein kinase (MAPK) signaling pathway, which mediates the phosphorylation of mechanistic target of rapamycin (mTOR) and the transport of ATG9, thus promoting the initiation of autophagy. Mitophagy is mainly mediated by BCL2/adenovirus E1B 19kDa interacting protein 3/Nip3-like protein X, and Parkin/PINK1 (PTEN induced putative kinase 1) (22). Radiation induced the accumulation of mitochondrial ROS, which resulted in mitochondrial damage that was in turn recognized by Parkin (23).

### 2.2 Immunogenic cell death

Immunogenic cell death (ICD), known as immunogenic apoptotic cell death or immunogenic apoptosis, has been defined as a “form of regulated cell death that is sufficient to activate an adaptive immune response in immunocompetent hosts” (24). Cellular redistribution and extracellular release of DAMPs are the main features and mechanisms of ICD, including CRT, HSPs, HMGB1, adenosine 5-triphosphate (ATP), spliceosome-associated protein 130, defensins and S100 proteins.

IR-induced OS can induce immunogenic death of tumor cells through multiple mechanisms (25). (1) Endoplasmic reticulum (ER) stress, caused by overproduction of ROS, has been shown to be responsible for ICD (26–30). CRT is overexpressed and released during ER stress. IR-mediated ER stress produces DAMPs molecules via PERK and IRE1-α phosphorylation, especially CRT, acting as an ‘eat me’ signal to stimulate the antigen presenting function of dendritic cells, acting as an ‘eat me’ signal to stimulate the antigen presenting function of DCs (31). Currently, various therapies, including photodynamic therapy, hyperthermia, and nanomaterials have been explored as ways to enhance ER stress to promote ICD (26, 32, 33). (2) Mitochondria is another important pathway of ROS production induced by IR. Increased ROS level can be directly detected by receptor-interacting protein 1 and leading to autophosphorylation on serine residue 161 (34). And then recruits RIP3 and induces the formation of a functional necrosome with pore formation and the release of DAMPs (35, 36). After IR, mitochondrial DNA is oxidized in tumor cells, which will be phagocytosed by DCs and activate the STING-TBK1-IRF3-IFN-β pathway in DCs, which subsequently cross-presented irradiated tumor cell-derived antigens to CD8^+^T cells and elicited antitumor immunity (37). (3) Lysosome membrane permeability (LMP): After the radiation-induced ROS were perceived by the cells, LMP changed, which would trigger further downstream approaches to induce ICD (38). After the lysosomal membrane is destroyed, cathepsin D and the prozymogenic form of caspase 8 are released into the cytoplasm (39). Caspase 8 induces ER-associated BAP31 cleavage through downstream caspase activation and cleaves the BH3-only protein Bid into its truncated form, t-Bid, which promotes CRT expression on the cell surface by inducing mitochondrial outer membrane permeation and anterograde ER-Golgi traffic (39–41).

### 2.3 Ferroptosis

Ferroptosis is another way of radiation-induced tumor cell death, which is caused by lipid peroxidation caused by iron metabolism disorder and ROS accumulation. Lipid peroxidation is an important marker of ferroptosis. IR-induced ROS can remove electrons from polyunsaturated fatty acids (PUFAs) to form fatty acid free radicals (PUFA•), which rapidly react with molecular oxygen to form lipid peroxyls (PUFA-OO•) and form lipid hydroperoxides (PUFA-OOH) through Fenton reaction, which lead to lipid peroxidation of membrane phospholipids, eventually resulting in ferroptosis (42). In addition, IR-induced acyl-CoA synthetase long-chain family member 4 (ACSL4) expression increased PUFA-PL biosynthesis, which together with ROS drove PUFA-PL peroxidation (PUFA-PL-OOH) and ferroptosis (43, 44).

**Figure 3:**
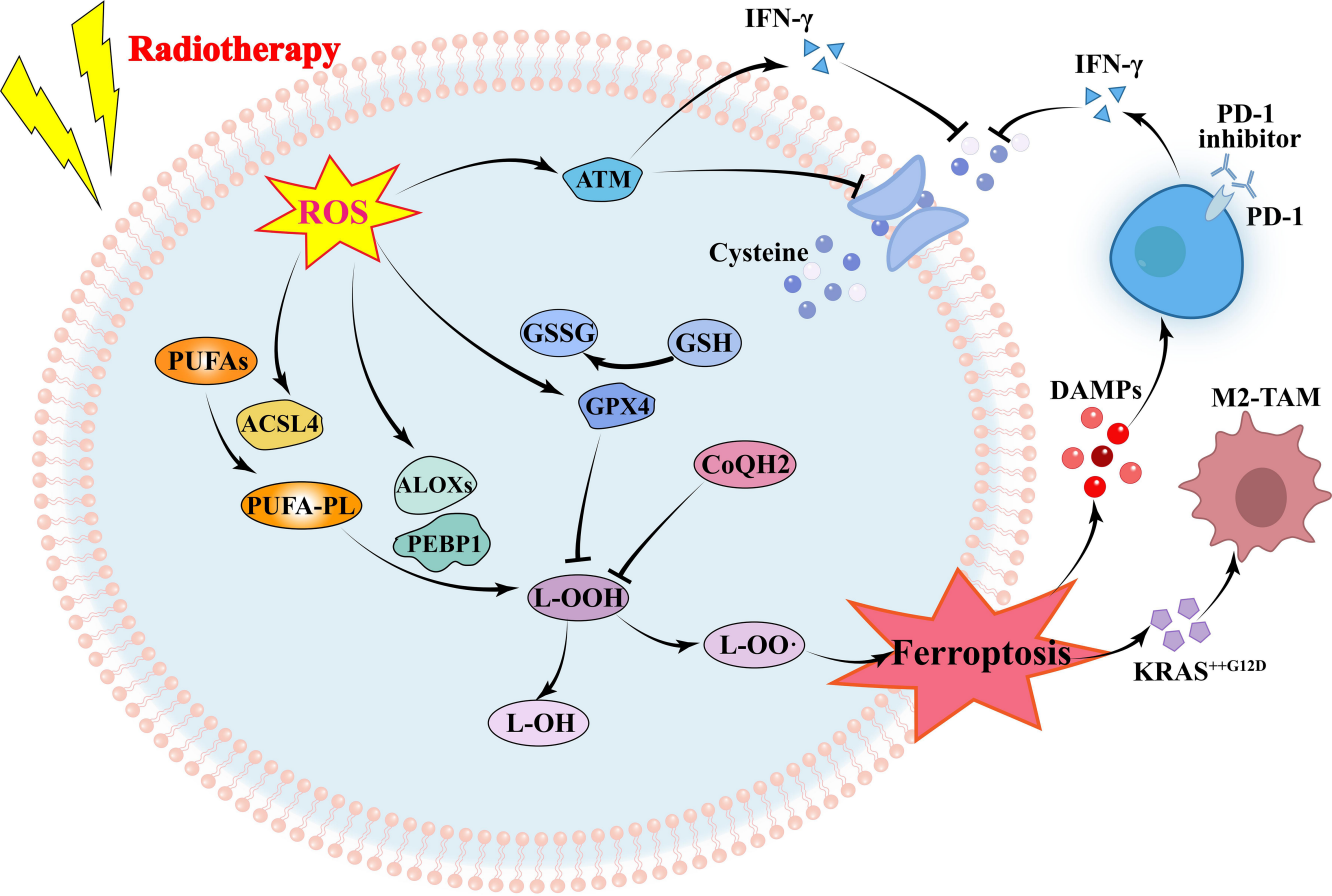
Mechanism of radiotherapy and immunotherapy in regulating ferroptosis of tumor cells. RT-induced ROS increases ferroptosis by increasing lipid peroxidation and decreasing glutathione. Immunotherapy and RT-activated T cells secreting IFN-γ inhibit glutathione synthesis by reducing glutamine transport, to promoting ferroptosis. ROS, reactive oxygen species; ATM, ataxia telangiectasia mutated protein; IFN, interferon; GSH, glutathione; GSSG, Glutathione Oxidized; GPX4, glutathione peroxidase 4; PUFAs, polyunsaturated fatty acids; ACSL4, acyl-CoA synthetase long-chain family member 4; ALOXs, Lipoxygenases; PEBP1, Phosphatidylethanolamine-binding Protein 1; DAMPs, damage-associated molecular patterns; TAM, tumor-associated macrophage.

Ferroptosis is an important way of tumor cell death induced by RT, and lipid peroxidation induced by OS is the basis of ferroptosis. RT not only induced ROS production, but also induced the expression of ACSL4, a lipid metabolizing enzyme required for ferroptosis, leading to elevated lipid peroxidation and ferroptosis (44). IR-induced ROS attack PUFAs to produce lipid peroxides (L-OOH), resulting in the death of iron. The peroxidation process is mediated by lipoxyphenase and phosphatidyl ethanolamine binding protein 1. L-OOH can be reduced to the corresponding alcohol by glutathione peroxidase or by panthenol by Fenton reaction L-OO (45–47). Recombinant solute carrier family 7, member 11 (SLC7A11 or xCT) can promote glutathione (GSH) synthesis and reduce L-OOH production to reduce the occurrence of ferroptosis (48). Radiation activates the ataxic-telangiectasia mutant (ATM), which inhibits SLC7A11 expression and promotes ferroptosis (49). It has been suggested that tumor ferroptosis is a new intersection between RT and adaptive immune response. IFN-γ released by immunotherapy-activated CD8^+^T cells down-regulates the expression of SLC3A2 and SLC7A11, inhibits cystine uptake in cancer cells, and enhances ferroptosis specific lipid peroxidation in tumor cells (50). Nanoparticle loaded miR-21-3p increases ROS production by directly targeting thioredoxin reductase 1, thereby enhancing lipid peroxidation to promote IFN-γ-mediated ferroptosis and acting synergistically with anti-PD-1 antibodies (51). Radiation-induced ATM activation is also an important source of IFN-γ, which synergies with IFN-γ derived from CD8^+^T cells to inhibit SLC7A11 and increase ferroptosis in tumor cells (52). In an oxygen-deficient environment, neutrophils are able to transfer granules containing myeloperoxidase into tumor cells, thereby inducing the accumulation of lipid peroxides and iron in tumor cells and triggering ferroptosis (53). On the other hand, immune cells in the TME also undergo ferroptosis. CD36 mediates the uptake of fatty acids by tumor-infiltrating CD8^+^T cells, induces lipid peroxidation and ferroptosis, and leads to reduced cytotoxic cytokine production and reduced anti-tumor ability (54). Since Tregs are more resistant to ROS-induced ferroptosis, therapeutic strategies that can specifically induce lipid peroxidation and ferroptosis accumulation in Tregs need to be developed (55). Ferroptosis is more likely to occur in M2 TAM than M1 (56). Hsieh et al. (57) found that zero-valent iron (ZVI), a nanoparticle capable of inducing ferroptosis in tumor cells, was able to convert M2 type TAM into M1 type, lifting the inhibition of anti-tumor immunity. However, in pancreatic cancer, OS was able to induce secretion of KRASG12D-containing exosomes, which were taken up by macrophages to mediate autophagy-dependent ferroptosis and switch TAM to the M2 phenotype (58). In addition, in TME, the expression of proteins associated with ferroptosis and lipid peroxidation in natural killer cells (NKs) was increased, suggesting that the dysfunction of NKs may be related to ferroptosis (59). Similarly, ferroptosis in DCs decreases antigen presentation and antitumor ability, and prevents CD8^+^T cells from producing IFN-γ (60). RT is often used in combination with ICIs, but lacks the expected survival benefit in some tumors. In this subset of patients, combining ferroptosis inducers with immunotherapy and RT may be a good strategy for enhancing tumor ferroptosis and sensitizing such tumors to immunotherapy and RT. Drugs have also been developed to promote ferroptosis, inducing ferroptosis while also enhancing the immune response caused by immune checkpoint inhibitors (61). However, Due to the contradictory effects of ferroptosis on tumor cells and immune cells, the clinical application value of ferroptosis in immune cells needs further exploration (**Figure 3**).

## 3 OS regulates TME

### 3.1 OS regulates immune checkpoint expression

IR is considered as a precipitating factor of immune checkpoint expression (62, 63), and ROS plays a key role in this process (64). In macrophages, treatment with ROS inducers resulted in increased expression of PD-L1 (65). Mechanistically, ROS accumulation activated nuclear factor kappa-B (NF-κB) signaling to promote PD-L1 transcription and immunosuppressive chemokine release. In pancreatic cancer, ROS can also induce the expression of fibroblast growth factor receptor 1, which can directly induce the expression of PD-L1 or by activating protein kinase B (AKT) signaling (66). ROS induced by IR is one of the main causes of DNA double-strand breaks (DSBs). DSBs activate ATM kinase, which is able to convert signals to ataxia telangiectasia and Rad3-related (ATR), thereby upregulating checkpoint kinase 1 (Chk1) activity to promote PD-L1 expression, mediated through STAT1/3-IRF1 pathway (67). The fragment DNA produced by DSBs can also be sensed by intracellular cGAS, thereby activating STING-TBK1-IRF3 signal to increase the secretion of type I IFN, which is an important way to induce the expression of PD-L1 (68, 69). Similarly, during OS in mitochondria, oxidized mitochondria DNA is released into the cytosol and then it induces IFN signaling via cGAS-STING-TBK1, which upregulates PD-L1 and IDO-1 expression to inhibit T-cell activation (16). In addition to PD-L1, ATR-Chk1 activation also contributes to integrin-associated protein (CD47) expression, which is mediated by STAT3 (70). CD47 is another immunoglobulin highly expressed on the surface of tumor cells, which can inhibit macrophage-mediated phagocytosis by binding to its major ligand signal regulatory protein α, thereby negatively regulating anti-tumor immunity (71). However, it has also been reported that ROS can also down-regulate the expression of CD47, which is mediated by hypoxia-inducible factor 1α (HIF-1α) (72, 73).

### 3.2 OS regulates immune cells

**Figure 4:**
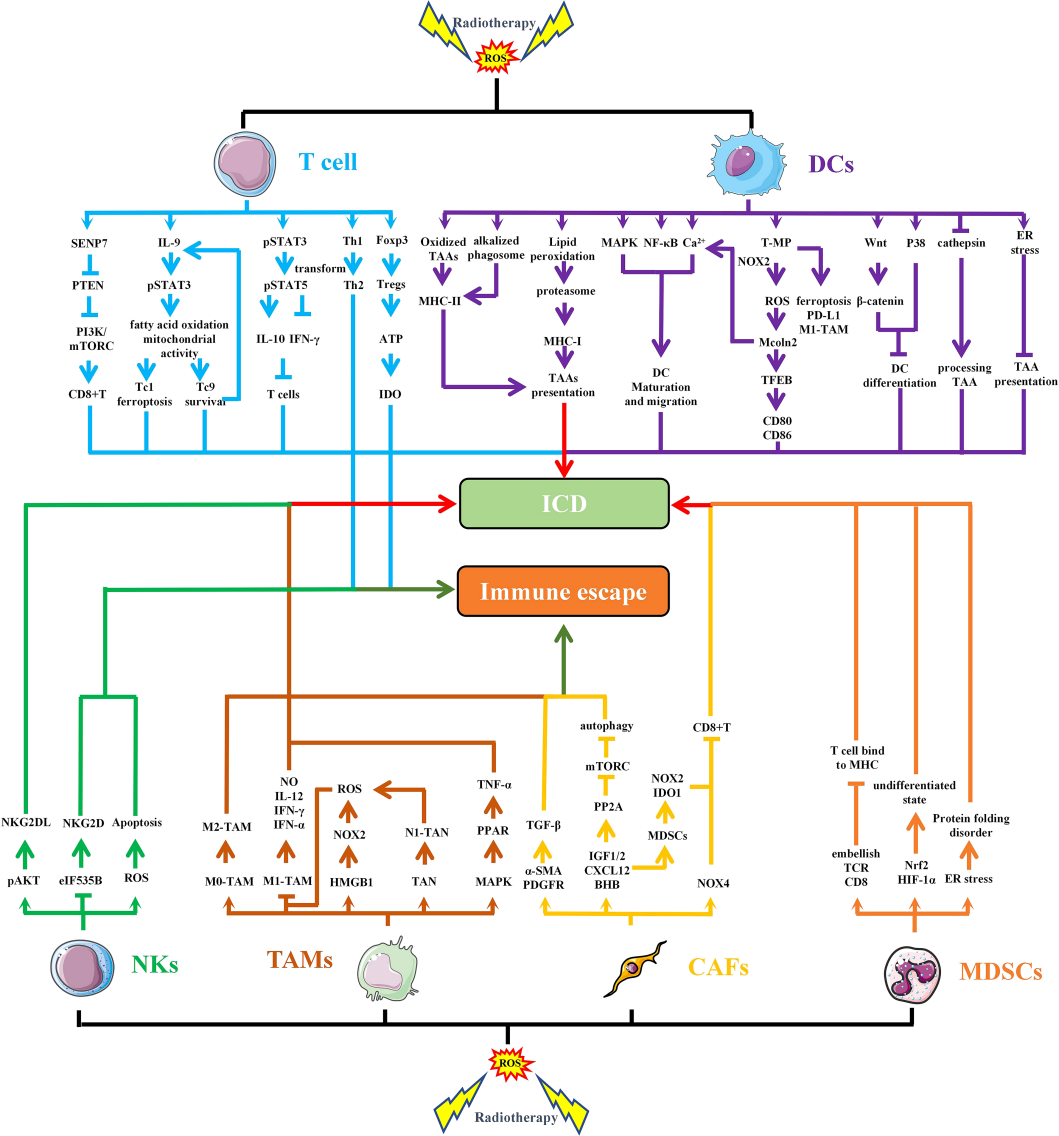
RT-induced ROS regulates the function and activation of immune cells. IR-induced ROS regulates the function of different cells in TME through multiple pathways, leading to immunogenic death or immune escape of tumor cells. SENP7, SUMO-specific protease 7; PTEN, phosphatase and tensin homolog deleted on chromosome 10; PI3K, Phosphoinositide-3 kinase; IL, interleukin; STAT, signal transducer and activator of transcription; IFN, interferon; CD, cluster of differentiation; Foxp3, forkhead box protein 3; ATP, adenosine 5-triphosphate; IDO, indoleamine 2,3-dioxygenase; MHC, major histocompatibility complex; TAAs, tumor-associated antigens; MAPK, mitogen-activated protein kinase; NF-κB, nuclear factor kappa-B; T-MP, tumor cell-derived microparticles; NOXs, NADPH oxidases; EB, transcription factor EB; ICD, immunogenic cell death; NO, nitrous oxide; TANs, Tumor-associated neutrophils; TNF, tumor necrosis factor; PPAR, peroxisome proliferator activated receptor; TGF, transforming growth factor; α-SMA, α-smooth muscle actin; PDGFR, platelet—derived growth factor receptor; PP2A, protein phospholipase; IGF, insulin like growth factor; CXCL, chemokine (C-X-C motif) ligand; BHB, β-hydroxybutyrate.

IR-induced ROS also regulates the function and activation of immune cells (74). In TME, several major immune cells, such as T cells, Tregs, NKs, DCs, TAMs and MDSCs, are regulated by OS (**Figure 4**). Among T cells, CD8^+^T cells are the main specific immune killer cells that perform tumor killing. Of course, T cells also include many helper T cells. NKs are the main innate anti-tumor immune cells, which can directly kill tumor cells. DCs is the primary antigen presenting cell responsible for delivering TAAs to other immune killer cells. TAMs are divided into M1 type and M2 type, in which M1 type is an inflammatory phenotype and contributes to anti-tumor, while M2 type has the effect of promoting tumor immune escape. MDSCs and Tregs are the two main cells that inhibit anti-tumor immunity, and their increase often indicates immune escape of tumor cells.

#### 3.2.1 T cells

ROS concentration is a necessary precursor for the activation and function of CD8^+^T cells, especially during TCR signal transduction after tumor antigen stimulation (75). It is well known that phosphatase and tensin homolog deleted on chromosome 10 (PTEN) is an important inhibitory molecule in the PI3K/AKT/mTOC pathway and has immunosuppressive function. SUMO-specific protease 7 (SENP7), a reversible posttranslational modification protease, can sense OS to induce PTEN degradation, thereby maintaining metabolic fitness and effector functions of CD8^+^T cells (76). CD8^+^Tc9 (cytotoxic T lymphocyte subset 9) cells exert greater persistence and antitumor efficacy than Tc1 cells. Tc9 cell-derived IL-9 activated STAT3, upregulated fatty acid oxidation and mitochondrial activity, and rendered Tc9 cells with reduced lipid peroxidation and resistance to ROS-induced ferroptosis in the TME (77). The increase of ROS significantly down-regulates the expression of miR-155-5p in tumor cells. Targeting miR-155-5p may be an effective method to improve TME by down-regulating PD-L1 and increasing infiltration of CD8^+^T cells (78). Appropriate level of ROS is a key signaling molecule involved in directing T cell activation and differentiation. However, with too high level, it inhibits proliferation, damages DNA, and induces apoptosis (79). It has been suggested that elevated ROS is a driver of T cell depletion. ROS elevation drives elevated nuclear factor of activated T cells localization and sustained signaling, inducing depleted T cell phenotypes with elevated PD-1, T cell immunoglobulin domain and mucin domain-3, and T cell immunoreceptor with Ig and immunoreceptor tyrosine-based inhibition motif domains (80). In addition, ROS induced decreased IFN-γ secretion in T cells and increased IL-10 production through the STAT3-STAT5 axis (81). ROS can also cause changes in Th1/Th2 polarity, which is conducive to the production of more Th2 cells, which is conducive to the tumor (82). NF-κB is believed to be the key factor in inhibiting T cell function and inducing T cell death induced by OS (79). Chronic or persistent low-dose OS down-regulates NF-κB in naive and mature activated/memory T cells, and the synergistic effect of Ros-mediated NF-κB suppression and elevated TNF-α level leads to T cell death (83).

Tregs are recruited into the TME to mediate immunosuppression and are susceptible to OS in the TME (84). Cellular OS state induces and maintains the epigenetic modification, transcription, translation, and post-translational stability of FoxP3, the master regulator of Tregs (85). In TME with OS, Tregs are able to compete with DCs and effector T cells for cysteine, the feedstock of GSH, resulting in inhibition of DCs function and downregulation of T cell activation and proliferation (86). In addition, the weak Nrf2-related antioxidant system in Tregs transforms them into apoptotic Tregs in oxidized TME, which can self-supply and release ATP, and convert it to adenosine to mediate, sustain, and amplify powerful suppression in the TME (84)

#### 3.2.2 DCs

DCs are fundamental for the initiation and maintenance of immune responses against cancer cells and have been shown to generate ROS during antigen presentation (87, 88). Immature DCs mainly have the ability to migrate, while mature DCs can mediate antigen presentation. During the differentiation of monocytes into DCs, expression of the DNA repair proteins XRCC1, ligase IIIα, poly (ADP-ribose) polymerase-1, and catalytic subunit of DNA-dependent protein kinase become up-regulated, making DCs repair-competent and ROS-resistant (89). Oxidized antigens have higher immunogenicity, which is reflected in enhanced MHC-II antigen processing and presentation by DCs (90). ROS can promote maturation of DCs and promote antigen presentation through phagosomal alkalinization (87). In DCs, ROS production and lipid peroxidation promote the escape of antigens from endosomes into the cytoplasm so that the proteasome can be degraded into peptides and processed into MHC I (91). In addition, transient intracellular ROS rapidly induced cytosolic mobilization of Ca^2+^, differential activation of mitogen-activated protein kinases, and nuclear translocation of NF-κB, which activate immature DCs to mature and potently enhance migration (92). DNA fragments generated by IR can promote the production of cGAMP, which can enter the TME and be ingested by DCs, activating STING signal (37, 93). STING-dependent cytosolic DNA sensing in DCs initiates antitumor immune responses, OS as a metabolic regulator promotes STING-mediated DCs antitumor immune responses and highlights SENP3 as an overflow valve for STING signaling induction in the metabolically abnormal TME (94). Tumor cell-derived microparticles (T-MP) increase lysosomal pH via NADPH oxidase 2 (NOX2, previously known as gp91*^phox^*)-catalyzed ROS production, promoting the formation of MHC class I-tumor antigen peptide complexes (95). It has been reported that radiation and chemotherapy can produce microparticles from tumor cells. Irradiation-induced T-MP can be taken up by tumor cells and TAMs, resulting in increased ferroptosis, PD-L1 expression, and M1 phenotype TAMs (96). Moreover, T-MP increased ROS activates the lysosomal Ca^2+^ channel Mcoln2, leading to Ca^2+^ release and activation of the transcription factor EB, a lysosomal master regulator that directly binds to CD80 and CD86 promoters and promotes gene expression, which facilitates antigen presentation (95). However, ROS has also been reported to have harmful effects on DCs. High glucose medium impaired the differentiation of monocytes to DCs by inducing ROS to activate Wnt/β-catenin and p38 MAPK (97). Overproduction of ROS also inhibits the hydrolytic function of cathepsin in DC, thus inhibiting antigen processing (98). Meanwhile, chronic activation of endoplasmic reticulum stress responses induced by excess ROS inhibits the ability of DC to present antigens to T cells (99, 100). Mitochondrial metabolism in plasmacytoid DCs (pDCs) which acquire the cross-presentation ability after stimulation by toll-like receptor (TLR) ligands is important to the induction of adaptive immune responses. The reduction of mitochondrial ROS production dramatically decreases the cross-presentation capacity of pDCs, leading to a strong reduction of their capacity to trigger CD8^+^T cells responses (101).

#### 3.2.3 NKs

NKs are lymphocytes capable of eliminating tumor cells without prior sensitization to a specific antigen (102). However, in TME, ROS is quite detrimental to NK survival and function. ROS induced in NK promotes NK cell apoptosis, while activation of antioxidant pathway increases NK resistance to OS (103, 104). Inhibition of NOx2-derived ROS in combination with cell-activating cytokines or checkpoint inhibitors can enhance NK and T cell function (105). NKs-expressed activating receptor Natural Killer Group 2D (NKG2D) acts as a “master switch” in controlling the arousal state of NK cells. NKG2D-mediated cytotoxicity is related to the level of NKG2D ligands (NKG2DLs) expressed by tumor cells. It has been reported that ROS-induced AKT phosphorylation induces the expression of NKG2DLs, which would sensitize tumor cells to NKs (106). In breast cancer, ROS reduces translation-initiation rate-limiting factor (eIF535B) phosphorylation at Ser2 and down-regulates NKG2D expression to inhibit NK cell activity (107).

#### 3.2.4 TAMs and tumor-associated neutrophils

In the tumor microenvironment, TAMs mainly include M1 and M2 phenotypes, among which M1 type has highly pro-inflammatory and anti-tumor functions, while M2 type contributes to suppress immune response. ROS plays a critical role in the differentiation of TAMs (108). M1 macrophages are more sensitive to OS, so ROS can polarize M0 macrophages into a tolerant M2 phenotype (109). It has also been reported that ROS is a critical trigger of cyclooxygenase-2–mediated macrophage differentiation from monocytes (108). Inhibition of ROS could transform TAMs into M1 phenotype, promote the secretion of inducible nitric oxide synthase, IL-12, IFN-γ and TNF-α (110). HMGB1 is a key molecule in irradiation induced ICD. A study has confirmed that in hepatocellular carcinoma, HMGB1 binds to TLR2 on the surface of TAMs to produce ROS via NOX2 (111). The increase of ROS promotes an increase in autophagy, which polarizes TAMs toward the M2 phenotype. In melanoma, ROS is also capable of ectopic peroxisome proliferator activated receptor-γ from the nucleus into the cytoplasm via mitogen-activated protein kinase kinase 1, thereby inducing an increase in TNF-α, which not only plays an antitumor role, but also converts TAMs into a phenotype that promotes tumor invasion (112).

Tumor-associated neutrophils (TANs) play an important role in TME. Irradiation can activate NOXs in neutrophils and produce ROS, which can increase phagocytosis (113). TANs include both antitumor (N1) and proto-tumor (N2) phenotypes, in which N1 TAN can be induced by RT. RT-induced N1 TAN produced more ROS, leading to tumor cell apoptosis and subsequent activation of tumor-specific cytotoxic T lymphocytes (CTLs) (114).

#### 3.2.5 MDSCs

There is a close relationship between the immunosuppressive function of MDSCs and ROS. ROS molecules are essential for maintenance of MDSCs in their undifferentiated state. Scavenging ROS induces differentiation of immature myeloid cells into macrophages, while differentiate into macrophages and DCs in the absence of NOX activity (115, 116). In polymorphonuclear MDSC, ROS-induced ER stress is the main factor driving MDSC function. ROS accumulation can induce oxidative modification of ER coelomins and thus affect protein folding (117, 118). Clearing ROS significantly attenuates MDSC immunosuppression by inhibiting the unfolded protein response. In addition, the release of ROS by MDSCs is one of the main mechanisms used to inhibit human T cells. ROS produced and secreted by MDSCs can modify TCR and CD8 molecules in T cells, resulting in CD8^+^T cells losing the ability to bind MHC (119). Furthermore, beyond their role in MDSC-mediated immune-suppression, ROS molecules are intrinsically involved in activation of transcription factors such as Nrf2 and HIF-1α, which can induce transcriptional and metabolic reprogramming of MDSCs and influence their differentiation and maintenance (9).

#### 3.2.6 Cancer-associated fibroblasts

Cancer-associated fibroblasts (CAFs) are key stromal cells in TME and promote tumor growth by releasing various growth factors. Myofibroblasts (an activated form of fibroblast) express smooth muscle actin (alpha-SMA) and platelet-derived growth factor receptors after IR, activating TGF-β signaling and angiogenesis via mitochondrial ROS to promote tumor growth (120). In addition, Insulin-like growth factor-1/2(IGF1/2), chemokine (C-X-C motif) ligand 12(CXCL12), and beta-hydroxybutyrate produced by CAFs can induce autophagy of cancer cells after RT, and promote recovery of irradiated cancer cells and tumor regeneration after RT. These CAFs-derived molecules, IGF1/2, CXCL12 and beta-hydroxybutyrate, increased ROS level after radiation, thereby enhancing protein phosphatases 2A activity, inhibiting mTOR activation, and increasing autophagy in cancer cells (121). CAFs are thought to promote immune escape through a variety of mechanisms and are regulated by ROS induced by NOX4 (122). NOX4 inhibition is capable of promoting CD8^+^T cell infiltration within tumors and restoring the immunotherapeutic response of CAFs-rich tumors. CAFs secrete CXCL2, facilitating the migration of monocytes to the local TME in lung squamous cell carcinoma and promoting monocyte-to-MDSCs differentiation. CAFs-induced MDSCs further inhibit CD8^+^T cells proliferation by upregulating the expression of NOX2 and IDO 1 to generate excessive ROS (123).

## 4 Regulating OS to enhance the efficacy of immunotherapy

Given the effect of OS on TME, regulation of OS is considered an effective protocol for enhancing immunotherapy efficacy (**Table 1**).

**Table 1:** Potential approachs to modulating oxidative stress to enhance radiotherapy in combination with immunotherapy.

Classification Criteria	Treatment	Tumors	Characteristics
Induced ROS production	Au-OMV+RT	Glioblastoma	Increasing intracellular ROS, promoting MCP-1 and MCP-2, to increase chemotaxis of macrophages.
	Imiquimod+RT	Melanoma	Accelerating autophagy through MAPK and NF-κB signaling pathways, increasing CD4 and CD8+T cell ratios through IFN-γ and TNF-α production.
	Hb@Hf-Ce1 nanoparticles+radiotherapy‐radiodynamic therapy	Melanoma Colorectal cancer Mammary carcinoma	activating the photosensitizer Ce6 to produce ROS, inducing a comprehensive anti-tumor immune response.
	G-CSF+RT	Prostate carcinoma Mammary carcinoma Thymoma	stimulating ROS release of N1 TAN, enhancing the phagocytosis of macrophages.
	Anlotinib+ anti-PD-L1	Colorectal Cancer	Anlotinib could activate ROS/JNK/ AP-1 signaling pathway to increase the expression levels of PD-L1, IFN-α/β/γ, and CXCL2.
Photodynamic therapy	TBP-nMOF+αPD-1+PDT	Breast cancer	The efficient ROS production at low oxygen concentration, restoring the activity of suppressed CTLs in TME.
	nMOFs(W-TBP)+PDT	Breast cancer	Delivering immunostimulatory CpG oligodeoxynucleotides to DCs, enhancing the release of TAAs and the maturation of DCs.
	NCP@pyrolipid+PDT	Colorectal cancer	producing large amounts of ROS, blocking PD-L1, increasing CD8^+^T cell infiltration.
	VES-CSO/TPGS-RGD+PDT	Colorectal cancer	blocking PD-L1, increasing DCs maturation and ICD
Nanomedicine	Cu-NCPs+RT	Colorectal cancer	Eliminatiing GSH and producing ROS, augmenting radioimmunotherapy and T-cell infiltration
	M-FDH+RT	Breast cancer	Producing a 10.98-fold tumor oxygenation, causing efficient production of ROS, resulting in over 90% elimination of CAFs, enhancing CD8^+^T cells.
	MGTe+RT	Breast cancer	Inducing ROS production, up-regulating the ratio of M1 macrophages and levels of multiple cytokines.
	HfMOF-PEG-FA+RT	Colorectal cancer	increasing radiation dose deposition and production of ROS, promoting IFN regulatory factor stimulation, enhancing immune activation.
	RAN-PC+RT	Breast cancer	Enhancing CD8^+^T cells and IFN-γ expressing subtype, notably reduced immunosuppressive cells.
	LipC6	liver tumors	Inhibiting ROS production in TAM, promoting the polarization of the M1 phenotype, reducing the inhibition of CD8^+^T cells.
Ameliorate hypoxia to induce OS	Hyperbaric oxygen therapy	Multiple malignancies	blocking HIF1-α, reducing adenosine production, enhancing CD8^+^T cells, reducing Treg cell infiltration, decreasing expression of PD-L.
	PLGA-R837@Cat+RT+αCTLA-4	Breast cancer Colorectal cancer	Relieving the tumor hypoxia, inducing strong immune memory, increasing TAAs.

### 4.1 Induced ROS production

The histopathological feature of glioblastoma is the significant invasion of microglia and peripheral macrophages (the primary innate immune cells of the central nervous system) resident in the tumor. Researchers have designed a compound Au-OMV using gold nanoparticles and bacterial outer membrane vesicles to increase intracellular ROS production (124). ROS induced OS promotes the expression of macrophage/monocyte chemoattractant protein-1 (MCP-1) and MCP-2. The combination of Au-OMV with RT increases chemotaxis of macrophages and radiation sensitivity in glioblastoma. TLR7 agonist imiquimod binding RT-induced ROS accelerates autophagy in melanoma through MAPK and NF-κB signaling pathways and increases CD4 and CD8^+^T cell ratios through IFN-γ and TNF-α production (125). Alternol is a naturally occurring compound that promotes the production of DAMPs and pro-inflammatory cytokines through elevated ROS, thereby promoting CD8^+^T cell initiation and T cell infiltration in tumors (126). To improve the therapeutic efficacy of RT-radiodynamic therapy, an oxygen-rich X-ray nanoprocessor Hb@Hf-Ce1 nanoparticles were designed in which an encapsulated oxygen carrier (hemoglobin, Hb) was used to regulate oxygen balance in anoxic TME. In contrast, radioluminescence stimulated by Hf under X-ray IR can activate the photosensitizer Ce6 to produce ROS, thus inducing a comprehensive anti-tumor immune response (127). G-CSF can stimulate the release of neutrophils and monocytes and enhance the phagocytosis of macrophages. It could amplify the tumor-specific immune response induced by RT, which is mediated by ROS production from N1 TAN stimulated by G-CSF (114). Anti-angiogenic drugs have the effect of normalizing blood vessels, which will improve tumor hypoxia and increase ROS production after radiotherapy (128). Immunotherapy also has this effect, so anti-angiogenic drugs (bevacizumab) can also increase the therapeutic efficacy of immune checkpoint inhibitors (129). Anlotinib is another antiangiogenic drug, and in colorectal cancer, Anlotinib benefits the anti-PD-L1 immunotherapy by activating ROS/JNK/AP-1 pathway to upregulate PD-L1 expression (130).

### 4.2 Photodynamic therapy

Photodynamic therapy (PDT) uses photosensitizers (PSs) to absorb certain wavelengths of ultraviolet light and produce free radicals or ions, which in turn produce ROS to suppress tumors (131). Importantly, PDT may well initiate ICD. First, PDT promotes the transfer of CRT from the endoplasmic reticulum to the surface of cell membranes and enhances the expression of HSP, providing “eat me” signals (132). In addition, PDT-induced ROS can promote the secretion of various cytokines (IFN-γ, IFN-α, etc.) by immune cells, and promote the infiltration of APCs and CD8^+^T cells (133). Many preclinical studies have found beneficial antitumor effects of PDT in combination with immunotherapy. In one study, Zhang et al. developed phenyporphyrin-based nMOFs, and PSs (TBP) combined with αPD-1 to restore the activity of suppressed CTLs in immunosuppressive TME (134). In another study, Lin et al. constructed a cationic nMOF (W-TBP) that is highly loaded with the anion CpG, which enhances the release of TAAs and effectively enhances the maturation of DCs (135). In addition, a self-assembled nano-coordination polymer (NCP) nanoparticle loaded with oxaliplatin induced an increase in CD8^+^T cell infiltration in both primary and remote tumors blocking PD-L1 and production of large amounts of ROS (136). Another temophen-supported nanodrug delivery system (VES-CSO/TPGS-RGD) has been shown in colorectal cancer to promote PD-L1 expression via HIF-1α and synergistically increase DCs maturation and ICD with PD-L1 blocking (137). Conventional PDT is limited in clinical application by the depth of light penetration, while X-rays can break shallow penetration limits as an energy source (138). In turn, ROS produced by PDT can increase DNA damage and lipid peroxidation damage, thus enhancing RT sensitivity (139). This suggests that the development of PDT is a potential sensitization method for radiotherapy combined with immunotherapy.

### 4.3 Nanomedicine

A variety of nanomaterials have been developed to amplify OS-induced ICDs during RT. Cu-based nanoscale coordination polymers (Cu-NCPS) with mixed-valence (Cu/Cu^2+^) have been shown to trigger GSH elimination and ROS production, to augmented radiotherapy combined with immunotherapy and T-cell infiltration (140). Hypoxic TME limits ROS production during RT. A microvesicle-inspired oxygen-delivering polyfluorocarbon nanosystem loading DiIC18(5) and halofuginone (M-FDH) produced a 10.98-fold enhancement of tumor oxygenation and caused efficient production of ROS upon radiation, which resulted in over 90% elimination of CAFs, enhancement of CD8^+^T cells, and elimination of suppressive immune cells (141). A hybrid nanoplatform (designated as MGTe) composed of tumor- and bacteria-derived immunomodulators and nano-radiosensitizer was acted as a radiosensitizer to enhance the efficacy of RT by ROS generation and ICD (142). MGTe-induced ROS would up-regulate the ratio of M1 macrophages and level of multiple cytokines. The hafnium foundation organic framework (HfMOF-PEG-FA) modified with folate increases radiation dose deposition and subsequent production of ROS, promotes IFN regulatory factor (IRF) stimulation, and in combination with the TLR7 agonist imiquimod enhances immune activation, characterized by an increase in CD8^+^ and proliferative T cells (143). A ROS-responsive albumin nanocomplex of anti-PD-L1 and cabazitaxel (RAN-PC) was designed to promote their intratumor delivery with controllable release capacity and potentiate radiation-mediated antitumor immunity for cancer therapy (144). RAN-PC+radiotherapy treatment produced a 3.61- and 5.10-fold enhancement in CD8^+^T cells and IFN-γ expressing subtype, respectively, and notably reduced versatile immunosuppressive cells. A nanoliposome loaded C6-ceremide (LipC6) was developed to inhibit ROS production in TAM to promote the polarization of the M1 phenotype and reduce the inhibition of CD8^+^T cells (110).

### 4.4 Ameliorate hypoxia to induce oxidative stress to enhance radiotherapy combined with immunotherapy

Hypoxia is a hallmark of solid tumors that reduces their susceptibility to radiation therapy and is a driver of immunotherapy resistance (145). Given the enormous benefits of targeting hypoxia for RT and immunotherapy, a number of regiments have been developed to ameliorate hypoxia to increase the efficacy of radiotherapy combined with immunotherapy, including increasing oxygen supply to tumors, inhibiting mitochondrial oxidative phosphorylation (OXPHOS), inhibiting HIF activity (146). Hyperbaric oxygen therapy can be used to counteract the anoxic effect in the TME to achieve the large number of ROS produced after IR, which can improve the efficacy of RT (147). In addition, hyperoxia blocks the hypoxia-HIF1α-adenosine pathway to reduce adenosine production, thereby enhancing CD8^+^T cells and reducing Treg cell infiltration. Meanwhile, oxygen-depleted MDSC inhibits antigen-specific and nonspecific T cell activity via HIF-1α, which can be inhibited by hyperoxygen, leading to decreased expression of PD-L1 (147). Core-shell nanoparticle based poly (lactate-glycolic) acid (PLGA) was prepared by encasing water-soluble catalase (Cat) to decompose H2O2 into O2, and imiquimote (R837), a toll-like receptor-7 agonist, was loaded into the shell. The formed PLGA-R837@Cat nanoparticles modulate the immunosuppressive TME by relieving tumor hypoxia. TAAs produced after RT induce intense ICD with the help of PLGA-R837@Cat. Combined with CTLA-4 checkpoint blocking, tumor metastasis is effectively inhibited through a powerful distant effect (148).

## 5 Conclusion

Radiotherapy can induce DNA strand breaks, damage of large molecules such as lipids and proteins, and other reactions. ROS is considered to be an important cytotoxic and signaling molecule in these reactions, inducing oxidative stress response of tumor cells. With the combination of radiotherapy and immunotherapy, the effect of oxidative stress on immune response has gained attention. In fact, apoptosis, autophagy and ferroptosis induced by oxidative stress are related to immunogenic death of tumor cells. A variety of immune cells and immune checkpoints in the immune microenvironment are also regulated by ROS. Appropriate ROS is a necessary condition for the activation of some immune cells, but excessive ROS may be an important reason why many immune cells have difficulty in performing anti-tumor functions and become resistant to immunotherapy. Therefore, a variety of therapeutic approaches are being explored to regulate ROS production during radiotherapy to enhance the efficacy of combination radiotherapy. In the future, while focusing on the changes of oxidative stress in tumor cells, we still need to pay attention to the influence of ROS in the TME on immunotherapy.

## Author contributions

Conceptualization: XJ. Methodology: ZZ and JS. Software: XB. Validation: JX. Formal analysis: HW. Investigation: CB. Resources, XJ. Data curation: ZZ. Writing—original draft preparation: ZZ, JS, and CB. Writing—review and editing: XB, QZ, and XJ. Visualization: XJ. Supervision: XJ. Project administration: XJ. Funding acquisition: XJ. All authors contributed to the article and approved the submitted version.

## Glossary

**Table:**

RT	radiotherapy
ROS	reactive oxygen species
OS	oxidative stress
TAAs	tumor-associated antigens
TME	tumor immune microenvironment
IR	irradiation
DAMPs	damage-associated molecular patterns
HMGB1	high-mobility group box 1
HSPs	heat shock proteins
CRT	calreticulin
APCs	antigen-presenting cells
MHC	major histocompatibility complex
TCR	T cell receptor
STING	stimulator of interferon genes
cGAS	cyclic GMP-AMP synthase
cGAMP	cyclic GMP-AMP
DCs	dendritic cells
PD-L1	programmed cell death-ligand 1
MDSCs	myeloid-derived suppressor cells
IDO	indoleamine 2, 3-dioxygenase
TGF-β	transforming growth factor β
Tregs	regulatory T cells
TAM	tumor-associated macrophage
NKs	natural killer cells
FOXP3	forkhead box protein 3Nrf2, nuclear factor−erythroid 2 related factor 2
Bcl-2	B-cell lymphoma-2
ATG	autophagy protein MAPK, mitogen-activated protein kinase
mTOR	mechanistic target of rapamycin
ICD	immunogenic cell death
ATP	adenosine 5-triphosphate
ER	endoplasmic reticulum
LMP	lysosome membrane permeability
PUFAs	polyunsaturated fatty acids
PUFA•	fatty acid free radicals
ACSL4	acyl-CoA synthetase long-chain family member 4
L-OOH	lipid peroxides
SLC7A11 or xCT	solute carrier family 7, member 11
GSH	glutathione
ATM	ataxic-telangiectasia mutant
ZVI	zero-valent iron
NF-κB	nuclear factor kappa-B
AKT	activating protein kinase B
DSBs	DNA double-strand breaks
ATR	ataxia telangiectasia and Rad3-related
Chk1	checkpoint kinase 1
PTEN	phosphatase and tensin homolog deleted on chromosome 10
SENP7	SUMO-specific protease 7
NOX2	NADPH oxidase 2
pDCs	plasmacytoid DCs
NKG2D	Natural Killer Group 2D
TLR	toll-like receptor
CTLs	tumor-specific cytotoxic T lymphocytes
CAFs	cancer-associated fibroblasts
IGF1/2	insulin-like growth factor-1/2
CXCL12	chemokine (C-X-C motif) ligand
MCP-1	macrophage/monocyte chemoattractant protein-1
PDT	photodynamic therapy
PSs	photosensitizers
Cu-NCPS	Cu-based nanoscale coordination polymers
LipC6	nanoliposome loaded C6-ceremide
OXPHOS	oxidative phosphorylation
PLGA	poly (lactate-glycolic) acid
Cat	catalase
